# An improved authenticated key agreement protocol for telecare medicine information system

**DOI:** 10.1186/s40064-016-2018-7

**Published:** 2016-05-03

**Authors:** Wenhao Liu, Qi Xie, Shengbao Wang, Bin Hu

**Affiliations:** Hangzhou Key Laboratory of Cryptography and Network Security, Hangzhou Normal University, Hangzhou, 311121 China

**Keywords:** Authentication, Protocol, Biometrics, Smart card

## Abstract

In telecare medicine information systems (TMIS), identity authentication of patients plays an important role and has been widely studied in the research field. Generally, it is realized by an authenticated key agreement protocol, and many such protocols were proposed in the literature. Recently, Zhang et al. pointed out that Islam et al.’s protocol suffers from the following security weaknesses: (1) Any legal but malicious patient can reveal other user’s identity; (2) An attacker can launch off-line password guessing attack and the impersonation attack if the patient’s identity is compromised. Zhang et al. also proposed an improved authenticated key agreement scheme with privacy protection for TMIS. However, in this paper, we point out that Zhang et al.’s scheme cannot resist off-line password guessing attack, and it fails to provide the revocation of lost/stolen smartcard. In order to overcome these weaknesses, we propose an improved protocol, the security and authentication of which can be proven using applied pi calculus based formal verification tool ProVerif.

## Background

In Internet environment, especially in the C/S model, it is crucial to authenticate both the user and the server when the user needs to access services provided by the server (Khan et al. 2014). The telecare medicine information system (TMIS) has attracted great attention of researchers to establish a convenient communication over the Internet between patients at home and doctors at a clinical center or home health-care agency (Kaul and Awasthi 2013; Wen 2013). A doctor can easily get access to his patient’s medical history from TMIS, and diagnose quickly without repeating physical examination. Besides, TMIS can save the patients’ expenses and time (Xie et al. 2014). However, it is a great challenge to preserve the security and privacy of patient’s information transmitted over the Internet (Xie et al. 2013; Siddiqui et al. 2014).

### Related works

Wu et al. (2010) proposed the first two-factor authentication scheme for TMIS service. Since then, a lot of two-factor authentication protocols have been proposed (He et al. 2012; Wei et al. 2012; Zhu 2012; Muhaya 2015). He et al. (2012) showed that Wu et al.’s protocol could not resist insider attack and impersonation attack. And they gave an improved protocol using smartcard. However, Wei et al. (2012) showed that He et al.’s protocol failed to resist off-line password guessing attack, and they also proposed an improved scheme, but Wei et al.’s scheme has the same security defects. In order to fix the above drawbacks, Zhu (2012) proposed an improved scheme. Unfortunately, Zhu et al.’s scheme has been proven insecure by Muhaya (2015). Wu et al. (2012) proposed a password-based user authentication scheme for the integrated EPR information system. Later, Islam and Biswas (2014) found that Wu et al.’s (2012) scheme cannot resist privileged-insider attack, off-line password guessing attack and ephemeral secret leakage attack.

It’s an interesting topic to improve security and computation efficiency of the authentication schemes. Pu et al. (2010) designed an anonymous authentication scheme for TMIS service using the elliptic curve cryptography (ECC). Chen et al. (2012) proposed a dynamic-identity based authentication scheme for TMIS. However, Jiang et al. (2013) showed Chen et al.’s scheme (Chen et al. 2012) cannot withstand impersonation attack, off-line password guessing attack and denial-of-service attack. Recently, Xu et al. (2014) proposed a two-factor authentication key agreement protocol using ECC. Unfortunately, Islam and Khan (2014) showed that Xu et al.’s scheme (Xu et al. 2014) can neither withstand replay attack, nor provide the revocation of lost/lost smart or achieve strong authentication in login and authentication phases. In order to overcome the above defects, they proposed a new anonymous two-factor authentication protocol for TMIS. Recently, Zhang and Zhou (2015) pointed out that Islam et al.’s protocol has many security defects such as: (1) Any legal but malicious patient can reveal other user’s identity; (2) An attacker can launch off-line password guessing attack and the impersonation attack if he knows legal user’s identity. Zhang et al. then proposed a new ECC-based authenticated key agreement scheme in order to fix the above security problems. In 2015, Chaudhry et al. (2015) also showed that Islam et al.’s protocol (Islam and Khan 2014) suffers from user impersonation attacks and server impersonation attacks. And then they proposed an improved two-factor authentication protocol for TMIS. In fact, Chaudhry et al.’s scheme is insecure under lost/stolen smartcard disguised attack and off-line password guessing attack, for that an insider adversary can extract information (*r*_*i*_, *h*()) from the memory of the user’s smart card. As we generally use passwords which are low-entropy keys, the following attack is feasible in practice: suppose that $PW^{\prime }$ is the guessed password and *l*_*i*_ is the user’s identity, an insider adversary (e.g. a malicious server) can compute $l_{i}^{\prime } = h(ID_{i} ||PW^{\prime } ||r_{i} )$; if $l_{i}^{\prime } = l_{i}$, then the adversary successfully found the correct password *PW*_*i*_.

As biometric keys can maintain uniqueness property, they can neither be forged nor guessed easily. Therefore, biometric keys have been widely adpoted in authentication protocols. In 2010, Li and Hwang (2010) proposed a biometric based remote user authentication scheme using user’s biometric key to identify the correct user. Li et al. (2011) showed that Li and Hwang’s scheme is vulnerable to man-in-the-middle attack, and they proposed an improved biometrics-based remote user authentication scheme. However, Truong et al. (2012) pointed that Li et al.’s scheme cannot resist stolen verifier attack, reply attack and man-in-the-middle attack, and they proposed an improved remote user authentication scheme. However, the login and password change phase of their scheme is not efficient for practice. Later, Awasthi and Srivastava (2013) proposed a new robust biometrics-based remote user authentication scheme using smart cards in order to avoid the time-consuming exponential operations. Unfortunately, Dheerendra et al. (2014) demonstrated that Awasthi et al.’s scheme fails to resist online and off-line password guessing attack, and they proposed an improved biometrics-based authentication scheme for TMIS. In 2014, He and Wang (2014) proposed a robust multi-server authentication scheme using biometrics-based smart card. But Vanga et al. (2015) pointed that He and Wang’s scheme is vulnerable to a known session-specific temporary information attack and impersonation attack. And they proposed a secure biometrics-based multi-server authentication protocol using biometrics-based smart card, and provided simulation results of their scheme for the formal security verification using Automated Validation of Internet Security Protocols and Applications (AVISPA) tool (AVISPA; Lv et al. 2013).

### Our contributions

In this paper, we show that Zhang et al.’s protocol (Zhang and Zhou 2015) is vulnerable to lost/stolen smartcard disguised attack and off-line password guessing attack. And then we propose an improved protocol using biometric keys (fingerprint, face and palm-print, etc.) to resolve the security problems. Furthermore, we provide the simulation results of our scheme for the formal security verification, using applied pi calculus based formal verification tool ProVerif. Our protocol overcomes the weaknesses of Islam et al.’s scheme and Zhang et al.’s scheme, and has the similar efficiency in comparison with their schemes.

The rest of paper is organized as follows: we first review Zhang et al.’s protocol in second section, and show the security weaknesses of Zhang et al.’s protocol in third section. Then, we propose an improved authentication protocol for TMIS is in fourth section. The security analysis of the improved scheme is given in fifth section. We prove the session key secrecy and authentication property using pi calculus based ProVerif in sixth section. In seventh section, we compare security and computation cost between our scheme and other related schemes. We conclude the paper in eighth section.

## Review of Zhang et al.’s scheme

In this section, we review Zhang et al.’s scheme. There are two participants in Zhang et al.’s protocol, patient *U* and telecare server *S*. Table 1 shows the notations used in this paper.

**Table 1:** The notations

Notations	Description
*U*	Patient in TMIS
*S*	Telecare server in TMIS
*ID*	Patient *U*’s identity
*PW*	Patient *U*’s password
*s*	Telecare server’s secret key
*Q*_*s*_	Telecare server’s public key, where *Q*_*s*_ = *sP*
*E*_*k*_/*D*_*k*_	Symmetric encryption/decryption algorithm with key *k*
*H*(·)	Secure one-way collision-resistant hash function
||	String concatenation operation
⊕	Exclusive OR operation

### Initialization phase

*S* selects an elliptic curve *E*_*p*_(*a*, *b*) over a prime finite field *F*_*p*_ and a base point *P* over *E*_*p*_(*a*, *b*). Followed that, *S* chooses a random number $s \in Z_{p}^{*}$ as his secret value, and computes *Q*_*s*_ = *sP*, and selects a one-way hash function $H( \cdot ):\{ 0,1\}^{*} \to Z_{p}^{*}$, and publishes {*E*_*p*_(*a*, *b*), *P*, *H*(·), *Q*_*s*_} and keeps *s* as a secret value.

### Registration phase

1. *U* selects his identity *ID*, its password *PW* and a random number *r*, and computes *l* = *H*(*r*||*PW*) and sends (*ID*, *l*) to *S* via a secure way.
2. Upon receiving (*ID*, *l*), *S* verifies user’s legitimacy in his database. If *ID* is a new patient, *S* sets *N* = 0, otherwise, *U* is re-registering to the system, *S* sets *N* = *N* + 1, and stores (*ID*, *N*, *T*) into its database, where T is the current registration time.
3. *S* computes *σ* = *H*(*s* ⊕ *ID*), *v* = *σ* ⊕ *l*, *μ* = *H*(*ID* ⊕ *l*) and stores {*v*, *μ*, *P*, *H*(·), *N*, *E*_*p*_(*a*, *b*)} into the smart card, and sends it to *U* via a secure way.
4. On obtaining the smartcard, *U* stores the number *r* in it.

### Login and authentication phase

1. *U* inserts his smart card into the terminal and inputs his identity *ID* and password *PW*. The smartcard computes *l* = *H*(*r*||*PW*), $\mu^{\prime } = H(ID \oplus l)$, and checks whether $\mu^{\prime } = \mu$ holds. If not, it aborts the session; otherwise, it selects a random number *a* and a current timestamp *T*_1_. Then, smartcard computes *V* = *aP*, *I* = *aQ*_*s*_, $K_{u} = H(I||T_{1} )$, $\sigma = v \oplus l$, $D = H(V||N||\sigma )$ and $G_{1} = E_{{K_{U} }} (ID||D)$. Then, smartcard sends login information *m*_1_ = {*V*, *G*_1_, *T*_1_} to *U* via the public channel.
2. After receiving *m*_1_ at *T*_2_, *S* checks whether *T*_2_ − *T*_1_ < ∆*T* is valid. If it is true, *S* computes *I* = *sV*, $K_{s} = H(I||T_{1} )$, and decrypts *G*_1_ to get $ID^{\prime }$ and $D^{\prime }$, and checks if $ID^{\prime }$ is found in the database. If not, *S* terminates the session; otherwise, *S* computes $\sigma^{*} = H(s \oplus ID^{\prime } )$ and checks whether $D^{\prime } = H(V||N||\sigma^{*} )$ holds. If not, this session terminates; otherwise, *S* selects a random number *c* and computes *W* = *cP*, *J* = *cV*, $sk_{s} = H(ID^{\prime } ||I||J)$, $G_{2} = H(\sigma^{*} ||ID^{\prime } ||sk_{s} ||W||T_{2} )$, and *S* sends *m*_2_ = {*W*, *G*_2_, *T*_2_} to *U* via the public channel. If *T*_2_ is invalid, abort, otherwise, smartcard computes *J* = *aW*, $sk_{u} = H(ID||I||J)$, $G_{2}^{\prime } = H(\sigma ||ID||sk_{u} ||W||T_{2} )$, and checks whether $G_{2}^{\prime } = G_{2}$ holds. If not, it aborts the session; otherwise, *U* authenticates *S* successfully.

### Password updating phase

*U* inserts his smart card into the terminal and enter his *ID* and *PW* when he wants to update its password.

1. The smartcard computes $l = H(r||PW)$, $\mu^{\prime } = H(ID \oplus l)$, and checks whether $\mu^{\prime } = \mu$ holds. If not, it aborts the session; otherwise, it selects a new random number $r^{*}$ and a new password $PW^{*}$, and updates corresponding value in the smart card.
2. The smartcard computes $\sigma = v \oplus l$, $l^{*} = H(r^{*} ||PW^{*} )$, $v^{*} = \sigma \oplus l^{*}$, $\mu^{*} = H(ID \oplus l^{*} )$ and replaces (*v*, *μ*) with $(v^{*} ,\mu^{*} )$, respectively.

### Lost/stolen smartcard revocation phase

When *U*’s smartcard is lost or stolen, it will request *S* for its revocation.

1. *U* chooses its new password $PW^{*}$ and new random number $r^{*}$, and computes $l^{*} = H(r^{*} ||PW^{*} )$, and submits $(ID,l^{*} )$ to *S* over a secure channel.
2. *S* firstly checks the registration credentials of *U*. If the credential provided by *U* is valid, *S* updates *N* as *N* = *N* + 1 for the tuple (*ID*, *N*, *T*_1_) to revoke the smartcard.
3. *S* computes $\sigma = H(s \oplus ID)$, $v^{*} = \sigma \oplus l^{*}$, $\mu^{*} = H(ID \oplus l^{*} )$, and stores $\{ v^{*} ,\mu^{*} ,P,H( \cdot ),Q_{s} ,N,E_{p} (a,b)\}$ into the smart card, and sends it to *U* via a secure way.
4. On obtaining the smartcard, *U* stores the random number $r^{*}$ in it. Finally, the smartcard stores $\{ r^{*} ,v^{*} ,\mu^{*} ,P,H( \cdot ),Q_{s} ,N,E_{p} (a,b)\}$.

## Weaknesses of Zhang et al.’s scheme

Through careful analysis, we find that Zhang et al.’s protocol is vulnerable to off-line password guessing attack and lost/stolen smartcard disguised attack. The detailed analyses are described as follows.

### Off-line password guessing attack

If an insider adversary in TMIS can extract information (*r*, *μ*) from the memory of the user’s smart card (Zhang and Zhou 2015). Generally speaking, password is not high-entropy keys (Abadi and Fournet 2001). Therefore, the following attack is feasible in practice. Suppose that $PW^{\prime }$ is the guessed password, and an insider adversary (e.g. the user’s colleague or malicious server) may know the user’s identity easily.

The insider adversary in TMIS who knows *ID* can compute $l^{\prime } = H(r||PW^{\prime } )$, $\mu^{\prime } = H(ID \oplus l^{\prime } ) = H(ID \oplus H(r||PW^{\prime } ))$, and checks whether $\mu^{\prime } = \mu$ holds. If it is true, the insider adversary has guessed the correct password. Otherwise, it repeatedly guesses a new password until he succeeds.

### Failure to provide the revocation of lost/stolen smartcard

Though the Zhang et al.’s scheme has lost/stolen smartcard revocation phase, an insider adversary can still use the lost/stolen smartcard to pass through the authentication process. The reason is that $\sigma = H(s \oplus ID)$ and *ID* in the new smart card are the same as that of the lost/stolen smartcard, and *N* = *N* + 1, according to off-line password guessing attack, the adversary can easily get *PW* and compute the correct authentication request message *m*_1_ = {*V*, *G*, *T*_1_}, which can pass the authentication of the server.

## The improved scheme

In our improved scheme, $\{ s,E_{p} (a,b),P,H( \cdot ),Q_{s} \}$ are the same as that of Zhang et al.’s scheme.

### Registration phases

When a user *U* wants to become a legal user, he should register to *S* as follows.

1. *U* selects his identity *ID*, password *PW* and a random number *r*, and computes $l = H(r||PW)$, and sends (*ID*, *l*) to *S* via a secure way.
2. Upon receiving (*ID*, *l*), *S* verifies user’s legitimacy in his database. If *ID* is a new patient, *S* sets *N* = 0, otherwise, *U* is re-registering to the system, *S* sets *N* = *N* + 1, and stores the tuple (*ID*, *N*, *N*_*c*_) to its database, where *N*_*c*_ is the identity of the smart card.
3. *S* computes $\alpha = H(s \oplus ID)$, $\beta = \alpha \oplus l$ and stores $\{ \beta ,P,H( \cdot ),Q_{s} ,N,N_{c} ,E_{p} (a,b)\}$ into the smart card, and sends it to *U* via a secure way.
4. On obtaining the smartcard, *U* scans and enters his personal biometrics *Bio*. It is worth mentioning that no one can get *Bio* except *U* and the biometrics scanner can be combined in the smart card reader. *U* computes $\mu = r \oplus H(Bio)$, $\theta = H(ID||PW||r)$, *U* stores $(\mu ,\theta )$ in the smart card.

### Login and authentication phases

In this phase, the user *U* and the server *S* can be authenticated each other and establish the session key *sk*, which showed in Algorithm 1.

1. *U* inserts his smart card into the terminal and inputs his identity *ID*, password *PW* and *Bio*. The smartcard computes $r^{\prime } = \mu \oplus H(Bio)$, $\theta^{\prime } = H(r^{\prime } ||PW||ID)$, and checks whether $\theta^{\prime } = \theta$ holds. If not, it aborts the session; otherwise, it selects two random numbers *a* and *N*_1_. Then, smartcard computes *V* = *aP*, *I* = *aQ*_*s*_, $K_{u} = H(I||N_{1} )$, $\alpha = \beta \oplus l$, $\gamma = H(V,N,N_{1} ,\alpha ,N_{c} )$ and $G_{1} = E_{{K_{U} }} (ID||N_{1} ||\gamma ||N_{c} )$. Then, smartcard sends login information *m*_1_ = {*V*, *G*_1_, *N*_1_} to *S* via the public channel.
2. After receiving *m*_1_, *S* checks whether *N*_1_ is a fresh nonce or not. If it is true, *S* computes *I* = *sV*, $$K_{s} = H(I||N_{1} )$$, and decrypts *G*_1_ to get $$ID^{\prime }$$, *N*_*c*_, $$\gamma$$ and *N*_1_, and checks whether or not $$ID^{\prime }$$ is found in the database. If not, *S* terminates the session; otherwise, *S* computes $$\alpha^{*} = H(s \oplus ID)$$, $$\gamma^{*} = H(V,N,N_{1} ,\alpha^{*} ,N_{c} )$$, and checks whether $$\gamma^{*} = \gamma$$ holds. If is not true, *S* terminates the session; otherwise, it selects two random numbers *c* and *N*_2_ for computing *W* = *cP*, *J* = *cV*, $$K = H(J||N_{2} )\,G_{2} = E_{K} (Q_{s} ||N_{2} ),$$$$sk = H(ID^{\prime } ||Q_{s} ||I||J||N_{1} ||N_{2} )$$, and *S* sends *m*_2_ = {*W*, *G*_2_, *N*_2_} to *U* via the public channel. If *N*_2_ is not a fresh nonce number, abort, otherwise, smartcard computes *J* = *aW*, $$K = H(J||N_{2} )$$, and decrypts *G*_2_ to get *Q*_*s*_ and *N*_2_, and checks whether or not $$Q_{s}^{\prime } = Q_{s}$$ holds. If not, smartcard terminates the session; otherwise, *U* authenticates *S* successfully, and computes $$sk = H(ID||Q_{s} ||I||J||N_{1} ||N_{2} )$$.

**Algorithm 1** Login and authentication phases
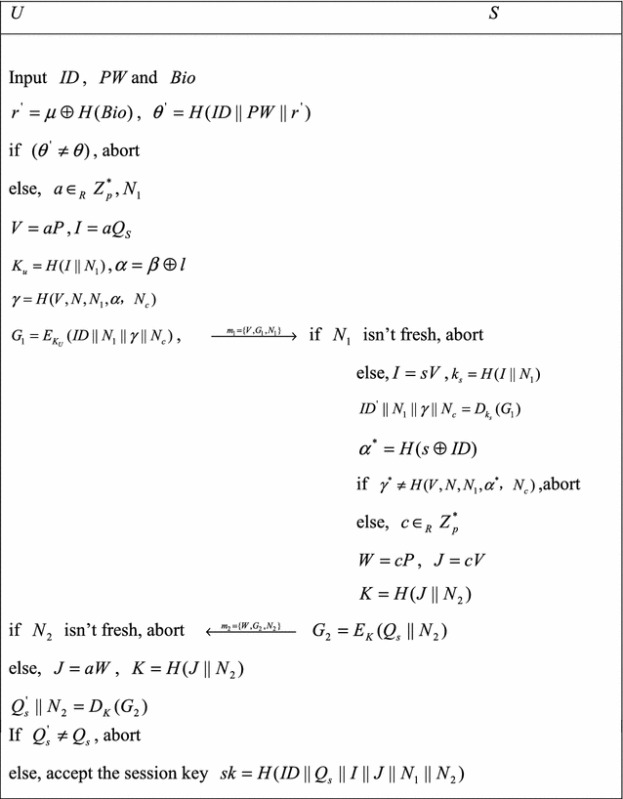


### Password updating phases

*U* inserts his smart card into the terminal and enter his *ID* and *PW* when he wants to update its password.

1. The smartcard computes $r^{\prime } = \mu \oplus H(Bio)$, $l = H(PW||r^{\prime } )$, $\theta = H(ID||PW||r^{\prime } )$ and checks whether $\theta^{\prime } = \theta$ holds. If not, it aborts the session; otherwise, it selects a new random number $r^{*}$ and a new password $PW^{*}$, and updates corresponding value in the smart card.
2. The smartcard computes $\mu^{*} = r^{*} \oplus H(Bio)$, $\theta^{*} = H(ID||PW^{*} ||r^{*} )$ and replaces (*μ*, *θ*) with $(\mu^{*} ,\theta^{*} )$.

### Lost/stolen smartcard revocation phases

When *U*’s smartcard is lost or stolen, it will request *S* for its revocation.

1. *U* chooses its new password $PW^{*}$ and new random number $r^{*}$, and computes $l^{*} = H(r^{*} ||PW^{*} )$, $\mu^{*} = r^{*} \oplus H(Bio)$, $\theta^{*} = H(ID||PW^{*} ||r^{*} )$ and submits $(ID,l^{*} ,\mu^{*} ,\theta^{*} )$ to *S* over a secure channel.
2. *S* checks the registration credentials of *U*. If the credential provided by *U* is valid, *S* updates *N* as *N* = *N* + 1 for the tuple (*ID*, *N*, *N*_*c*_) to revoke the smartcard, and deletes *N*_*c*_ from his database and selects a new smartcard number *N*_*new*_ for *U*, and returns the tuple (*ID*, *N*, *N*_*new*_) to his database.
3. *S* computes $\alpha = H(s \oplus ID)$, $\beta^{*} = \alpha \oplus l^{*}$, $\theta^{*} = H(ID||PW||r^{*} )$, and stores $\{ \beta^{*} ,P,H( \cdot ),Q_{s} ,N,N_{new} ,E_{p} (a,b)\}$ into the smart card, and sends it to *U* via a secure way.
4. On obtaining the smartcard, *U* stores $(\mu^{*} ,\theta^{*} )$ in it. Finally, the smartcard stores $\{ \theta^{*} ,\mu^{*} ,\beta^{*} ,P,H( \cdot ),Q_{s} ,N,N_{new} ,E_{p} (a,b)\}$.

## Security analysis

In this section, we analyze the security of the improved protocol. The following attacks assume that a malicious adversary can eavesdrop, modify, insert, or delete any messages transmitted via public channel.

### The improved protocol can achieve mutual authentication

As *V* = *aP*, *I* = *aQ*_*s*_, $K_{u} = H(I||N_{1} )$, and $G_{1} = E_{{K_{U} }} (ID||N_{1} ||\gamma ||N_{c} )$, only the legal user *U* can get the secret value (*I*, *N*_1_) to generate a legal *G*_1_. *S* decrypts *G*_1_ and checks whether $ID^{\prime } = ID$ holds. If it is true, *S* can authenticate *U*, otherwise, *U* cannot be authenticated by *S*. On the other hand, *U* can authenticate *S* by verifying whether $Q_{s}^{\prime } = Q_{s}$ hold. As a result, our protocol achieves the mutual authentication.

### Malicious insider impersonation attack

Login phase: If a malicious user *U*_*A*_ wants to impersonate *U*, he must forge a valid login message $\{ V^{*} ,G_{1}^{*} ,N_{1} \}$ where $V^{*} = a^{*} P$, $I^{*} = a^{*} Q_{s}$, $K^{*} = H(I^{*} ||N_{1} )$, and $G_{1}^{*} = E_{{K^{*} }} (ID^{*} ||N_{1} ||\gamma ||N_{c} )$, however, *U*_*A*_ can not get *I*, such that it has to forge an invalid one. When *S* receives the login request message from *U*, it will decrypt and compute $G_{1}^{*} = E_{{K^{*} }} (ID^{*} ||N_{1} ||\gamma ||N_{c} )$, but the equation $ID^{*} = ID$ does not hold, therefore, *S* will reject the login request. Thus, our scheme can resist insider impersonation attack.

### Off-line password guessing attack

If a malicious attacker has stolen user’s smart card, then he can extract the information {*θ*, *μ*, *β*, *P*, *H*(·), *N*, *Q*_*s*_, *E*_*p*_(*a*, *b*)} from the smart card, where $\mu = r \oplus H(Bio)$, $\theta = H(ID||PW||r)$, $l = H(r||PW)$. Since *r* is protected by *Bio* and *PW* is protected by a one-way hash function, the attacker cannot know both of the real identity *ID* and the correct password *PW*. It is impossible to guess these two parameters correctly in polynomial time. Therefore, our protocol is secure against the off-line password guessing attack.

### Strong replay attack

If a malicious attacker wants to replay a previously transmitted message of the sender or the receiver, the attack will fail since *U* and *S* choose different random numbers (*N*_1_, *N*_2_) in each session. During the authentication phase, after *S* response the next login message $m_{1}^{\prime } = \{ V^{\prime } ,G_{1}^{\prime } ,N_{1}^{\prime } \}$ using a valid nonce *N*_1_, the attacker can neither verify its validness nor obtain the session key assuming the intractability of Diffie–Hellman problem.

### Lost/stolen smartcard attack

When the attacker attempts to insert the lost smart card into the device, it can’t pass the authentication of the server, since the stolen card’s *N*_*c*_ is updated in the database of *S*.

### Perfect forward secrecy

In our protocol, the session key is $sk = H(ID||Q_{s} ||I||J||N_{1} ||N_{2} )$, where *I* = *aQ*_*s*_ = *asP*, *J* = *cV* = *caP*. Since *a* and *c* are random numbers chosen by *U* and *S*, their values are changed in each session run. Therefore, our protocol can provide perfect forward secrecy.

## Formal verification

Some formal verification tools are used to prove the security of cryptographic protocols, such as BAN logic, AVISPA and ProVerif (Abadi et al. 2009). In this section, we prove the session key secrecy and authentication using formal verification tool ProVerif, which is based on applied pi calculus (Abadi and Fournet 2001). The reason is that ProVerif is performed automatically, and the errors can be detected easily, while the formal security proof is artificial structured, and the errors may not easy to be found.

The ProVerif code for the definition of functions, reduction, equation, free names and constants is as follows.
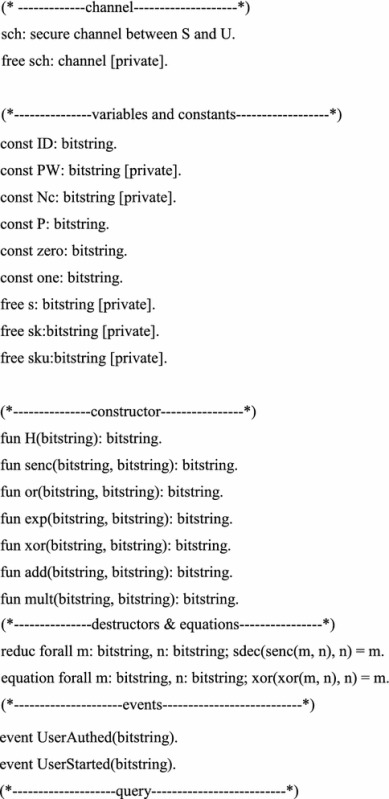

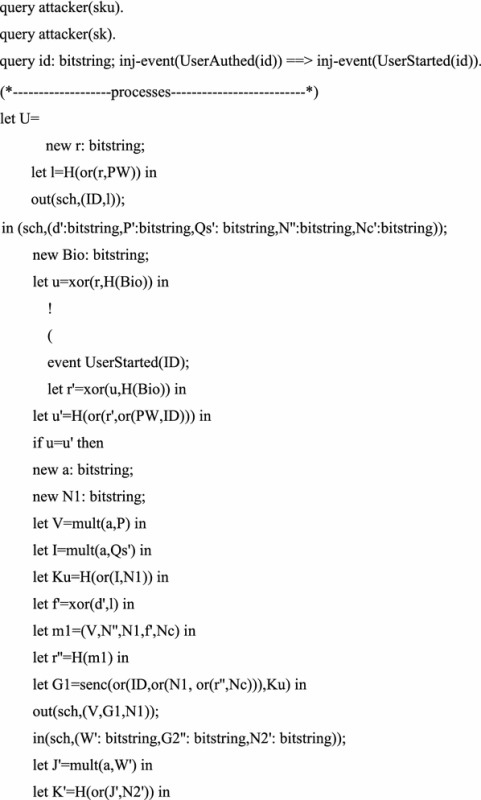

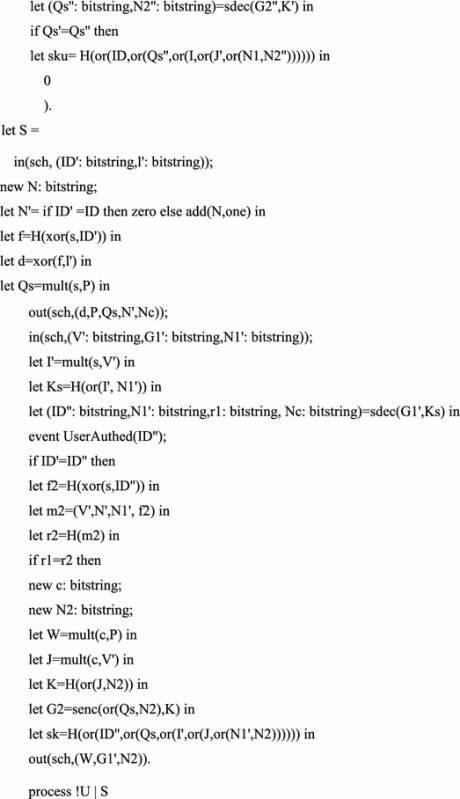


**Fig. 1:**
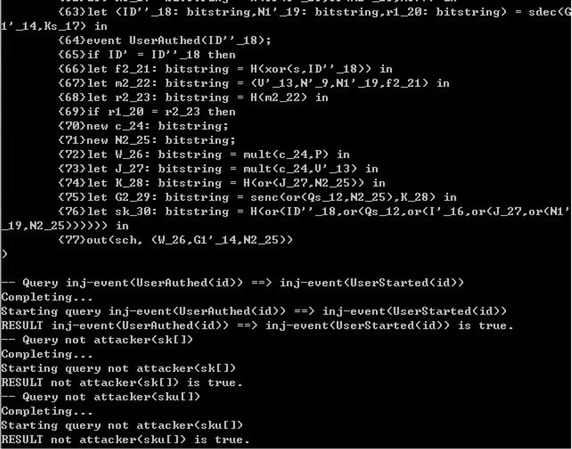
The performance result

We perform the above process in the latest version 1.88 of ProVerif. The performance results as shown in the Fig. 1. The experimental results show that our scheme is security.

## Security and computation cost comparisons

The security comparison between our scheme and other recently proposed related schemes are given in Table 2.

**Table 2:** Security comparison between our scheme and other schemes

Security attributes/schemes	Li and Hwang (2010)	Li et al. (2011)	Truong et al. (2012)	Awasthi and Srivastava (2013)	Dheerendra et al. (2014)	He and Wang (2014)	Vanga et al. (2015)	Ours
Provide user anonymity	N	N	Y	Y	N	N	Y	Y
Insider attack	N	Y	Y	Y	N	Y	Y	Y
Stolen smart card attack	Y	Y	Y	Y	N	Y	Y	Y
Replay attack	Y	Y	Y	Y	Y	N	Y	Y
Off-line password guessing attack	Y	Y	Y	N	Y	Y	Y	Y
Mutual authentication	N	Y	Y	N	Y	Y	Y	Y
Known session-specific temporary information attack	N	N	N	N	N	N	Y	Y
Perfect forward secrecy	N	N	N	N	N	Y	Y	Y
Impersonation attack	N	N	N	N	N	N	Y	Y
Provide lost smartcard revocation	N	N	N	N	N	N	Y	Y
Server spoofing attack	N	N	N	N	N	Y	Y	Y
Efficient login phase	N	N	N	Y	Y	Y	Y	Y
Efficient password change phase	N	N	N	N	Y	Y	Y	Y
Biometric update phase	N	N	N	N	N	Y	Y	Y

Let *T*_*m*_ be the time complexity of point multiplication in a group, *T*_*a*_ be the time complexity of point addition in a group, *T*_*s*_ be a symmetric key encryption/decryption operation and *T*_*h*_ be a one-way hash operation. Table 3 illustrates the average running times of some commonly used operations estimated by Kilinc and Yanik (2014), and shows that point multiplication in a group is slower than point addition, hash function and symmetric encryption/decryption operation.

**Table 3:** The running time of different operations

Operations	Point multiplication	Point addition	Hash function	Symmetric encryption/decryption
Time (ms)	2.226	0.0288	0.0023	0.0046

Since Islam et al.’s scheme (Islam and Khan 2014) and Zhang et al.’s scheme (Zhang and Zhou 2015) are more efficient than other schemes. Therefore, in this section, we only present the computation comparison between our scheme and Islam et al.’s and Zhang et al.’s schemes, and very recently proposed related schemes, which showed in Table 4. From Table 4, we can see that our protocol is almost efficient than that of Zhang et al.’s and Islam et al.’s schemes. However, our protocol overcomes the weaknesses of Islam et al.’s and Zhang et al.’s schemes.

**Table 4:** Computation cost comparison in login and authentication phase

	Islam and Khan (2014)	Zhang and Zhou (2015)	Chaudhry et al. (2015)	Vanga et al. (2015)	Ours
Computational cost	6*T*_*m*_ + 1*T*_*a*_ + 10*T*_*h*_	6*T*_*m*_ + 2*T*_*s*_ + 11*T*_*h*_	7*T*_*m*_ + 8*T*_*h*_	5*T*_*m*_ + 3*T*_*s*_ + 13*T*_*h*_	6*T*_*m*_ + 4*T*_*s*_ + 11*T*_*h*_
Estimated time (ms)	13.4078	13.3905	15.6004	13.4767	13.3997

If the scheme can prevent the attack or satisfy the property, the symbol ‘Y’ is used. Otherwise, the symbol ‘N’ is used.

## Conclusion

In this paper, we have shown that Zhang et al.’s protocol cannot achieve some secure properties, including security against off-line password guessing attacks, and it fails to provide the revocation of lost/stolen smartcard. Technically, we adopt random numbers based authentication mechanism, instead of the timestamps that may cause time synchronization problem. An improved protocol is proposed in order to overcome those weaknesses. The simulation results show that when compared with existing protocols, our protocol provides the same level of efficiency and better security guarantees for TMIS applications.
